# Estimating the population at risk with soil transmitted helminthiasis and annual drug requirements for preventive chemotherapy in Ogun State, Nigeria

**DOI:** 10.1038/s41598-022-06012-1

**Published:** 2022-02-07

**Authors:** Hammed Oladeji Mogaji, Olatunji Olugoke Johnson, Abbas Bolaji Adigun, Oladunni Nimota Adekunle, Samuel Bankole, Gabriel Adewunmi Dedeke, Babatunde Saheed Bada, Uwem Friday Ekpo

**Affiliations:** 1 Department of Animal and Environmental Biology, Federal University, Oye-Ekiti, Ekiti, Nigeria; 2 Department of Pure and Applied Zoology, Federal University of Agriculture, Abeokuta, Nigeria; 3 Department of Mathematics, Manchester University, Manchester, UK; 4 Department of Research and Application, National Centre for Remote Sensing, Jos, Nigeria; 5 Department of Zoology and Environmental Biology, Olabisi Onabanjo University, Ago-Iwoye, Ogun Nigeria; 6 Department of Environmental Management and Toxicology, Federal University of Agriculture, Abeokuta, Nigeria

**Keywords:** Policy and public health in microbiology, Parasitic infection

## Abstract

Soil transmitted helminth (STH) infections are among the most common human infections worldwide with over 1 billion people affected. Many estimates of STH infection are often based on school-aged children (SAC). This study produced predictive risk-maps of STH on a more finite scale, estimated the number of people infected, and the amount of drug required for preventive chemotherapy (PC) in Ogun state, Nigeria. Georeferenced STH infection data obtained from a cross-sectional survey at 33 locations between July 2016 and November 2018, together with remotely-sensed environmental and socio-economic data were analyzed using Bayesian geostatistical modelling. Stepwise variable selection procedure was employed to select a parsimonious set of predictors to predict risk and spatial distribution of STH infections. The number of persons (pre-school ages children, SAC and adults) infected with STH were estimated, with the amount of tablets needed for preventive chemotherapy. An overall prevalence of 17.2% (95% CI 14.9, 19.5) was recorded for any STH infection. *Ascaris lumbricoides* infections was the most predominant, with an overall prevalence of 13.6% (95% CI 11.5, 15.7), while Hookworm and *Trichuris trichiura* had overall prevalence of 4.6% (95% CI 3.3, 5.9) and 1.7% (95% CI 0.9, 2.4), respectively. The model-based prevalence predictions ranged from 5.0 to 23.8% for *Ascaris lumbricoides*, from 2.0 to 14.5% for hookworms, and from 0.1 to 5.7% for *Trichuris trichiura* across the implementation units. The predictive maps revealed a spatial pattern of high risk in the central, western and on the border of Republic of Benin. The model identified soil pH, soil moisture and elevation as the main predictors of infection for *A. lumbricoides*, Hookworms and *T. trichiura* respectively. About 50% (10/20) of the implementation units require biannual rounds of mass drug administration. Approximately, a total of 1.1 million persons were infected and require 7.8 million doses. However, a sub-total of 375,374 SAC were estimated to be infected, requiring 2.7 million doses. Our predictive risk maps and estimated PC needs provide useful information for the elimination of STH, either for resource acquisition or identifying priority areas for delivery of interventions in Ogun State, Nigeria.

## Introduction

Soil transmitted helminth (STH) infections are among the most common human infections worldwide and have been classified as neglected tropical diseases by the WHO^1,2^. Most of the world’s STH infections are caused by four common parasitic helminths; *Ascaris lumbricoides* (roundworm), *Trichuris trichiura* (whipworm), *Necator americanus* and *Ancylostoma duodenale* (hookworms), whose life cycle are linked with the soil environment and a definitive human host^2,3^. STH infections are widely distributed in the tropical and subtropical regions, with the greatest numbers occurring in sub-Saharan Africa, the Americas, China and East Asia^1,3^. About 5 billion people are at risk, and 1.5 billion of the worlds’ population are currently infected^1,3^. Infections are predominantly abundant in areas characterized with poverty, favorable climate and lack of access to basic infrastructural amenities such as potable water supply, sanitation and hygiene facilities^4–9^.

Efforts targeted at controlling STH have been through preventive chemotherapy (PC), and involves large-scale administration of albendazole or mebendazole medicines with a major focus on school-aged children (SAC) in endemic communities^1,10^. The World Health Organization (WHO) recommends PC, either once a year (annually), when the baseline prevalence of infections is between 20 and 50%, or twice a year (biannually) when the prevalence is above 50%^10^. PC is expensive, costing donor agencies and developing economies billions of dollars^11^. For instance, since 2010, the World Health Organization (WHO) has coordinated the annual distribution of 600 million medicines for PC in endemic countries, with albendazole, donated by GlaxoSmithKline, and mebendazole, donated by Johnson & Johnson^12^.

In line with elimination goals, the WHO stipulates that endemic countries must consistently treat and protect at least 75% of its population that requires treatment. Globally, a total of 576 million (59.9%) of the estimated 1.1 billion children requiring albendazole or mebendazole medicines were treated in 2018, and about 71.3% of the implementation units (IUs) achieved the 75% effective coverage target for SAC^12,13^. However, the recently published 2020–2030 NTD road map, emphasized more specific targets by 2030 which includes (1) eliminating STH as a public health problem (< 2% proportion of moderate and heavy intensity infections) in 96 countries; (2) reducing by 50% the number of tablets required during PC for STH and (3) increasing domestic financial support for PC for STH, with 25 countries deworming children using domestic funds^12,14,15^.

These targets call for more refined commitments in the planning and delivery approaches for large-scale administration of PC tablets to SAC, most especially in areas where prevalence estimates are high^16–18^. It is therefore important to constantly delineate highly endemic areas using parasitological surveys, and produce risk maps for unsampled areas using geostatistical methods to support targeting and delivery of tablets for PC^19–21^. Approaches combining parasitological surveys, geographical information systems (GIS), remote sensing (RS), spatial and geostatistical analysis have been explored extensively to model and predict the risk of helminth infections on a national scale in China^20^, Cote’d voire^21^, Cambodia^22^, South America^23^ and Nigeria^24^. Yet, there is paucity of studies investigating risk factors explaining spatial distribution of STH at a more finite scale such as the implementation units (IUs). Model based geostatistical approaches are useful in understanding the variations that exist within IUs, which are necessary for targeting intervention in hotspot area with high prevalence.

Nigeria is endemic for all the four common STH infections, and leading in terms of burden, and the number of people infected in sub-Saharan Africa^25,26^. Information on the spatial distribution and risk of STH infections is needed to facilitate targeting of control efforts in the context of resource scarcity. The few published studies in the country, have used secondary survey data to produce county-level risk maps^24,27,28^ and annual drug requirements^24^. Since implementation of PC occur in a more finite scale at IUs (referred to as local government areas (LGAs) in Nigeria), we hypothesize that risk maps made at these levels using more recent parasitological data will offer more robust insight into disease distribution and risk factors associated with such distributions. In addition, data generated at such IUs would be useful to estimate number of people at risk or infected with STH, rounds of MDA and the drug requirements for PC.

We therefore present findings from a geostatistical analysis of soil-transmitted helminth infection data that were obtained from a state-wide community-based survey in Ogun State, Nigeria. The aims of this study were (1) to map and predict the spatial distribution of soil-transmitted helminth infections at 2 km spatial scale using a Bayesian geostatistical approach; (2) identify the most important climatic, environmental and socioeconomic determinants of soil-transmitted helminth infections (3) calculate the number of persons infected and; (4) estimate the annual drug requirements for preventive chemotherapy according to guidelines put forward by the World Health Organization (WHO).

## Methods

### Study area, design and population

**Figure 1:**
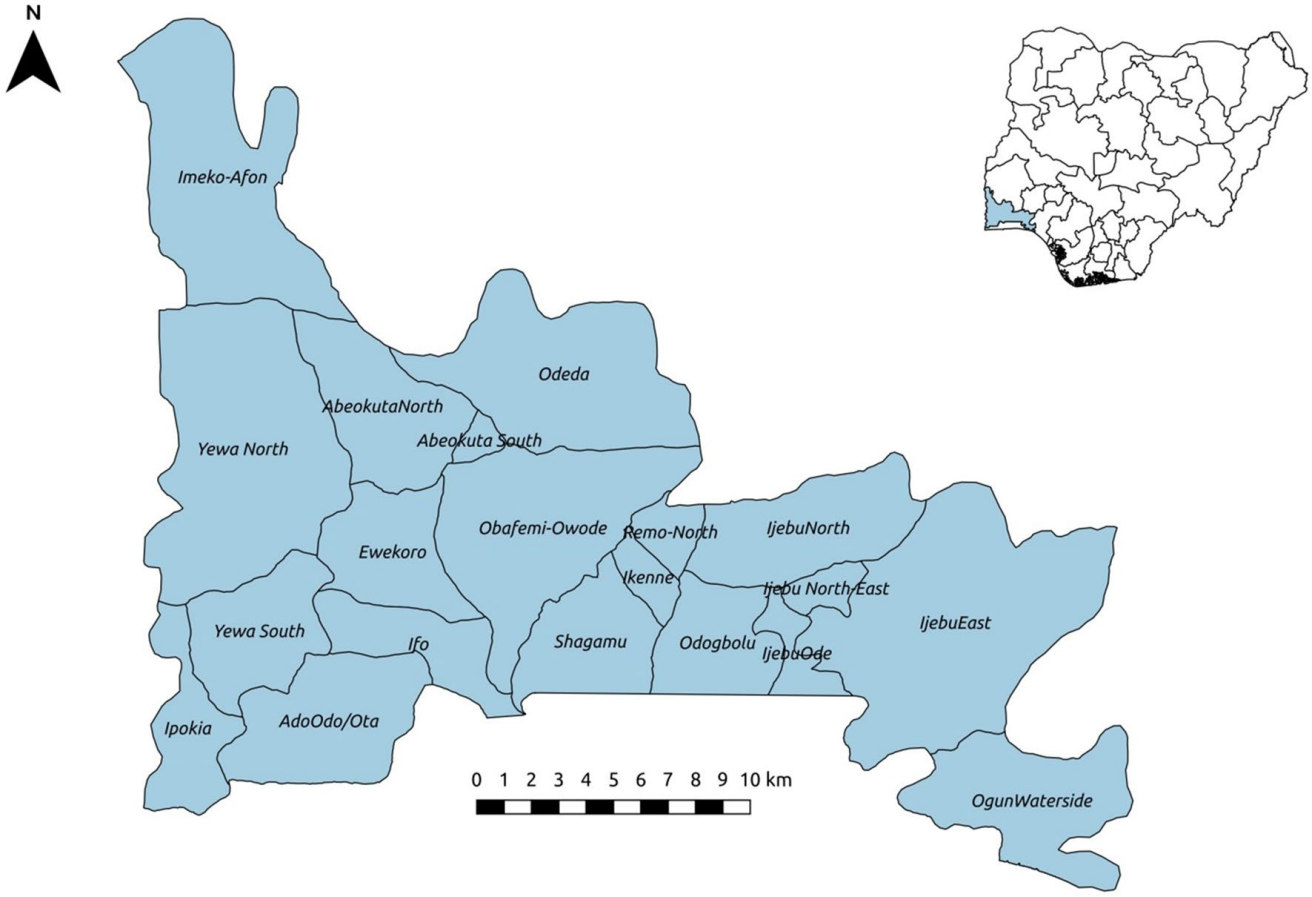
Map of the Ogun State showing the 20 implementation units (IUs) with Nigeria as inset. This figure was created by the authors in R programming software (R version 4.1.2, Vienna, Austria). Available at https://www.R-project.org/.

This study was conducted in Ogun State, Nigeria (Fig. 1). Details of the study area, design and population surveyed have been described elsewhere^19^. In brief, the study was carried out between July 2016 and November 2018 spanning across both wet and dry season. We designed a cross-sectional survey, and employed a systematic grid sampling method in the selection of communities to ensure an unbiased representation across the state^19^. A total of 1499 children and adults, from 33 spatially selected communities participated in the study^19^. In each community, all households and their occupants were considered eligible for participation and invited to participate in the study.

### STH Infection data

The field and laboratory procedures have been previously described in^19^. In brief, stool container was distributed to consenting household members in advance. Participants’ unique identifiers were marked on the containers and detailed instructions of how to collect a fresh morning stool sample were given. Stool samples were processed in a designated area provided by the community leader. Duplicate sediment slides were prepared from 1 g of each stool using SAF-Ether concentration method^19,29^. The slides were examined under a light microscope by experienced laboratory technicians 2 h post sample collection. Infection was defined as the presence of at least one helminth egg on one of the two slides. The parasites’ eggs were counted for each species, and number of eggs per species and per stool examined was recorded for each participant^19^.

### Environmental and socio-economic predictors

Nine environmental variables (elevation, enhanced vegetation index (EVI), normalized difference vegetation index (NDVI), land surface temperature for day (LSTD), land surface temperature for night (LSTN), rainfall, population, soil pH, and soil moisture; three socio-economic variables (night light emission (NLE), improved access to sanitation facilities and improved access to drinking water facilities) were used in the analysis. NLE was used as a proxy for urbanization and economic growth. These variables were chosen because they are either directly associated with prevalence of STH infections or they serve as proxy for other factors that are known to influence STH transmission^30^. All environmental and socio-economic data were obtained from open-access remote sensing data sources between 2016 and 2018 (Table 1). We resampled all the covariates to a spatial resolution of 2 km by 2 km using bilinear interpolation for continuous surface which is required to produce the spatial prediction of prevalence at every location in Ogun state.

**Table 1:** Data sources and properties of environmental and socio-economic covariates explored to model soil transmitted helminthiasis risk in Ogun State, Nigeria.

Data type	Source	Temporal resolution (days)	Temporal coverage	Spatial resolution (m)
**Environmental covariates**				
Elevation	WorldPop ^31^			100
Enhanced vegetation index (EVI)	MODIS/Terra ^32^	16	2016–2018	250
Normalized difference vegetation index (NDVI)	MODIS/Terra ^32^	16	2016–2018	250
Rainfall	MODIS/Terra ^32^	10	2016–2018	250
Soil pH	SoilGrids250m ^33^		2016–2018	250
Soil Moisture	C3S ^34^		2016–2018	1000
Population density	WorldPop ^31^		2017	100
Land surface temperature (Day)	MODIS/Terra ^32^	8	2016–2018	1000
Land surface temperature (Night)	MODIS/Terra ^32^	8	2016–2018	1000
**Socio-economic covariates**				
Night light emission	WorldPop ^31^		2017	100
Improved access to sanitation facilities	LBD ^35^		2017	5000
Improved access to drinking water facilities	LBD ^35^		2017	5000

### Population data

Population data of Nigeria at 100 m spatial resolution was obtained from World Pop^31^ population database. The total population count of people in Ogun state in 2017 was 5,103,988 out of 197,259,740 people in Nigeria. The number of school-aged children in Ogun state in 2017 was computed to be 1,913,868, which represents 37.5% of the total population of all persons.

### Variable selection procedures for the geostatistical model

To select the best set of covariates for the geostatistical model, we first examined the covariates for correlation using Pearson’s rank correlation index. Pairs of covariates with high correlation values (Pearson correlation > 0.7) were identified and only one of the correlated variables was included in the modelling process. The one included was chosen by visualizing the association via a scatterplot. Then we used both forward and backward stepwise selection to select a parsimonious set of covariates required for the prediction of STH among all the candidate set of covariates. This is achieved by fitting a non-spatial generalized linear model relating the prevalence of each STH species with the covariates. The final set of covariates result in a model with the lowest Akaike information criterion (AIC) and a further inclusion of any of the covariates does not improve the performance of the model.

### Geostatistical modelling

The prevalence survey data available for this analysis are, at any geographical location x, the number of individuals tested, m and the number of people tested positive for each of the STH species, $y_{k}$, where $y_{1}$ is the number of people that tested positive for Ascaris, $y_{2}$ for Hookworm, and $y_{3}$ for Trichuris. The sampling distribution of $y_{k}$ is binomial with number of trials m and probability of positive outcome $P_{k} \left( x \right)$, the prevalence at x. The variation of $P\left( x \right)$ was modelled using a combination of socio-economic and environmental covariate effects d(x); unexplained residual spatial variation, S(x). Therefore, we developed a binomial logistic geospatial model given as

$$log\left\{ {\frac{P\left( x \right)}{{1 - P\left( x \right)}}} \right\} = d^{\prime}\left( x \right)\beta + S\left( x \right).$$

$S\left( x \right)$ is modelled as a zero-mean discretely indexed Gaussian Markov Random Field (GMRF) defined on the triangulation of the domain of interest, such that the correlation between any two locations $x_{i}$ and $x_{j}$ is modelled using Matérn correlation function. S(x) serves two purposes in the model; (1) it helps to capture the geographical variation; and (2) it helps to predict prevalence at unobserved locations. The Matérn covariance function is given as

$$Cov\left( {S\left( {x_{i} } \right),S\left( {x_{j} } \right)} \right) = \frac{{\sigma^{2} }}{{2^{\nu - 1} \Gamma \left( \nu \right)}}\left( {\kappa u} \right)^{\nu } K_{\nu } \left( {\kappa u} \right),$$

where $d = x_{i} - x_{j}$, $\kappa$ is a scaling parameter, $K_{\nu }$ is the modified Bessel function of second kind and order $\nu > 0$ and $\Gamma \left( . \right)$ is the Gamma-function, $\sigma^{2}$ is the variance, and the spatial range $\rho = \sqrt 8 /\kappa$, the distance at which the spatial correlation is becomes negligible (< 0.1).

The model was fitted using the Integrated Nested Laplace Approximation (INLA) ^36,37^ and the Stochastic Partial Differential Equation (SPDE)^38^ approaches. INLAallows us to perform a fast Bayesian inference. Because no prior information was available, an independent vague zero-mean Gaussian prior distribution was assigned to the fixed and random effects parameters. Posterior distributions were obtained for all the parameters and were summarised to obtain the mean and 95% credible interval (CI). Prediction of the prevalence of each of the STH species were provided at 2 km spatial resolution throughout the study area. We then estimate the prevalence of any STH prevalence by assuming that each species is independent^38^. The predicted prevalence was represented as the posterior mean.

### Model validation

We validated our geostatistical model by assessing the predictive performance of the model using the 5-folds cross-validation. All survey data were randomly splitted into 5 groups. We hold-out each unique group then fit the model on the remaining groups and evaluate the predictive performance of the model on the hold-out group. The withheld data was matched with the predictions to summarize the performance of the model using the correlation, bias, root mean square error (RMSE) and the coverage probability**.**

### Estimating the amount of the anthelmintic treatment required

We estimated the amount of anthelmintic treatment (albendazole or mebendazole) needed to treat the population annually at the local government areas in Ogun state. According to the WHO STH treatment decision tree^39^, prevalence of STH should be examined after 5–6 rounds of annual or biannual PC. Subsequent chemotherapy campaigns after this evaluation should continue according to a set of endemicity classes defined by the following prevalence thresholds; suspend PC if prevalence is < 2%; biennial PC if prevalence is between 2 and 10%; annual PC if prevalence is between 10 and 20%; biannual PC if prevalence is between 20 and 50%; triannual PC is prevalence is greater than 50%^39^. Hence, we computed the total number of anthelmintic drugs by classifying each pixel according to the treatment decision, thereby estimating the number of MDA rounds. Then multiply the number of MDA rounds by the total population of that pixel. Hence, we aggregate across the pixels the number of anthelmintic drugs over the local government areas. Also, we estimated the number of anthelmintic drugs required to treat school-aged children (SAC) by multiplying the number of MDA rounds per pixel by the population of SAC per pixel. Then we constructed the local government level estimate by aggregating across the pixels.

### Estimating the number of people infected with STH

To estimate the number of people infected with STH parasites, we multiplied the prevalence at each pixel by the total population at that pixel. Also, to estimate the number of SAC infected with STH parasite, we multiplied the prevalence at each pixel by the population of SAC at that pixel. Hence, to construct the local government level estimate, we aggregate the values across the pixels. The 95% confidence interval of the estimate was constructed by using the prevalence values at the 2.5% and 97.5% quantiles.

### Ethical approval

Ethical clearance for this study (HPRS/381/183) was obtained from the Ethics review committee of Department of Planning, Research and Statistics, Ogun State Ministry of Health, Oke Imosan Abeokuta, Nigeria. Prior to data collection, visitations were made to the LGAs and the selected communities were the objectives and study procedures were explained and permissions for field survey were sought. Written informed consents were obtained from household heads and corresponding occupants of their households. Children below age sixteen, completed assent forms through their parents or guardians. All methods including recruitment of participants, collection of participant’s data and samples, laboratory analysis and data management were performed in accordance with the 1964 Declarations of Helsinki.

## Results

### Data summaries

A total of 1027 infection data was included in this survey. The demographic characteristics of the study population have been described elsewhere^19^. However, Table 2 summarizes the soil-transmitted helminths species-specific prevalence among the examined participants. In short, an overall prevalence of 17.2% (95% confidence interval (CI) 14.9, 19.5) was recorded for any STH infection. *Ascaris lumbricoides* infections was the most predominant, with an overall prevalence of 13.6% (95% CI 11.5, 15.7), while Hookworm and *Trichuris trichiura* had overall prevalence of 4.6% (95% CI 3.3, 5.9) and 1.7% (95% CI 0.9, 2.4), respectively. The geographical distribution of the empirical prevalence for each soil-transmitted helminth species has been described elsewhere^19^.

**Table 2:** Soil-transmitted helminth species-specific empirical prevalence across the 20 LGAs in Ogun State, Nigeria, Mid 2016-Early, 2018.

LGA	N	*Ascaris lumbricoides*		Hookworms		*Trichuris trichiura*		Any STH	
		n, %	95% CI	n, %	95% CI	n, %	95% CI	n, %	95% CI
Abeokuta South	14	5, 35.7	10.6, 60.8	1, 7.1	0, 20.6	0, 0	0, 0	6, 42.9	16.9, 68.6
Abeokuta North	40	7, 17.5	5.7, 29.3	1, 2.5	0, 7.3	0, 0	0, 0	8, 20	7.6, 32.4
Ado-Odo Ota	56	6, 10.7	2.6, 18.8	2, 3.6	0, 8.4	4, 7.1	0.4, 13.9	8, 14.3	5.1, 23.5
Ewekoro	46	3, 6.5	0, 13.7	0, 0	0, 0	0, 0	0, 0	3, 6.5	0, 13.7
Ifo	19	5, 26.3	6.5, 46.1	3, 15.8	0, 32.3	0, 0	0, 0	7, 36.8	15.2, 58.5
Ijebu East	38	6, 15.8	4.2, 27.4	0, 0	0	1, 2.6	0, 7.7	6, 15.8	4.2, 27.4
Ijebu Ode	25	0, 0	0, 0	0, 0	0, 0	0, 0	0, 0	0, 0	0,0
Ijebu North	48	6, 12.5	3.1, 21.9	1, 2.1	0, 6.1	0, 0	0, 0	6, 12.5	3.1, 21.9
Ijebu North-East	26	4, 15.4	1.5, 29.3	0, 0	0, 0	0, 0	0, 0	4, 15.4	1.5, 29.3
Ikenne	36	11, 30.6	15.5, 45.6	0, 0	0, 0	0, 0	0, 0	11, 30.6	15.5, 45.6
Imeko-Afon	86	4, 4.7	0.2, 9.1	2, 2.3	0, 5.5	0, 0	0, 0	5, 5.8	0.9, 10.8
Ipokia	13	4, 30.8	5.7, 55.9	2, 15.4	0, 35	3, 23.1	0.2, 46	5, 38.5	12, 64.9
Obafemi Owode	54	15, 27.8	15.8, 39.7	9, 16.7	6.7, 26.6	4, 7.4	0.4, 14.4	22, 40.7	27.6, 53.8
Odeda	71	4, 5.6	0.3, 11	6, 8.5	2, 14.9	0, 0	0, 0	8, 11.3	3.9, 18.6
Odogbolu	54	6, 11.1	2.7, 19.5	1, 1.9	0, 5.4	0, 0	0, 0	7, 13	4, 21.9
Ogun waterside	85	16, 18.8	10.5, 27.1	5, 5.9	0.9, 10.9	1, 1.2	0, 3.5	20, 23.5	14.5, 32.5
Remo North	15	4, 26.7	4.3, 49	2, 13.3	0, 30.5	0, 0	0, 0	6, 40	15.2, 64.8
Shagamu	128	9, 7	2.6, 11.5	3, 2.3	0, 5	0, 0	0, 0	10, 7.8	3.2, 12.5
Yewa North	114	3, 2.6	0, 5.6	2, 1.8	0, 4.2	1, 0.9	0, 2.6	6, 5.3	1.2, 9.4
Yewa South	59	22, 37.3	24.9, 49.6	7, 11.9	3.6, 20.1	3, 5.1	0, 10.7	29, 49.2	36.4, 61.9
	1027	140, 13.6	11.5, 15.7	47, 4.6	3.3, 5.9	17, 1.7	0.9, 2.4	177, 17.2	14.9, 19.5

N, number of samples examined; n, number of positives; CI, Confidence intervals.

### Geostatistical variable selection, model parameter estimates and model validation

Following the variable selection, Soil pH, soil moisture and elevation were selected for *Ascaris*, Hookworms, and *Trichuris* infections respectively (Table 3). The selected covariates were used to build predictive risk models specific to each of the three soil transmitted helminth species. A negative association exist between *Ascaris lumbricoides* infection and soil pH (odds ratio (OR) = − 0.05; 95% credible interval (CrI) − 0.071, − 0.029). Residual spatial correlation was estimated to be 12.813 km (95% CrI 5.199, 26.760 km). Similarly, a negative association was observed between Hookworms and soil moisture (odds ratio (OR) = − 0.027; 95% credible interval (CrI) − 0.042, − 0.015). Residual spatial correlation was estimated to be 17.823 km (95% CrI 4.834, 50.090 km). For *Trichuris trichiura*, infection risk was also negatively associated with elevation (odds ratio (OR) = − 0.027; 95% credible interval (CrI) − 0.042, − 0.015). Residual spatial correlation was estimated to be 5.427 km (95% CrI 2.789, 17.190 km). The predictive performance of the model based on a 5-folds cross-validation showed that; for *Ascaris lumbricoides*, the in-sample observed data and the predictions has a coefficient correlation of 0.82; a RMSE of 0.04; and a bias of − 0.005; and coverage probability of 0.86, respectively; for Hookworms, the in-sample observed data and the predictions has a coefficient correlation of 0.83; a RMSE of 0.06; and a bias of − 0.001; and coverage probability of 0.88, respectively; and for *Trichuris,* the in-sample observed data and the predictions has a coefficient correlation of 0.83; a RMSE of 0.07; and a bias of -0.01; and coverage probability of 0.89, respectively.

**Table 3:** Posterior estimates (median; 95% credible interval) of the final geostatistical models for soil-transmitted helminth infections in Ogun State, Nigeria in 2016–2018.

Species	Predictors	OR (95% CrI)
*Ascaris lumbricoides*	Intercept	− 0.051 (− 1.188, 1.014)
	Soil pH	− 0.051 (− 0.074, − 0.029)
	Spatial variance	0.861 (0.573, 1.240)
	Spatial scale (km)	12.813 (5.199, 26.760)
*Hookworm*	Intercept	− 1.323 (− 2.423, − 0.104)
	Soil moisture	− 0.027 (− 0.042, − 0.015)
	Spatial variance	0.918 (0.485, 1.540)
	Spatial scale (km)	17.823 (4.834, 50.090)
*Trichuris trichiura*	Intercept	− 3.028 (− 3.974, − 2.164)
	Elevation	− 0.027 (− 0.042, − 0.015)
	Spatial variance	1.158 (0.504, 2.290)
	Spatial scale (km)	5.427 (2.789, 17.190)

### Predictive risk maps of soil-transmitted helminth infections

**Figure 2:**
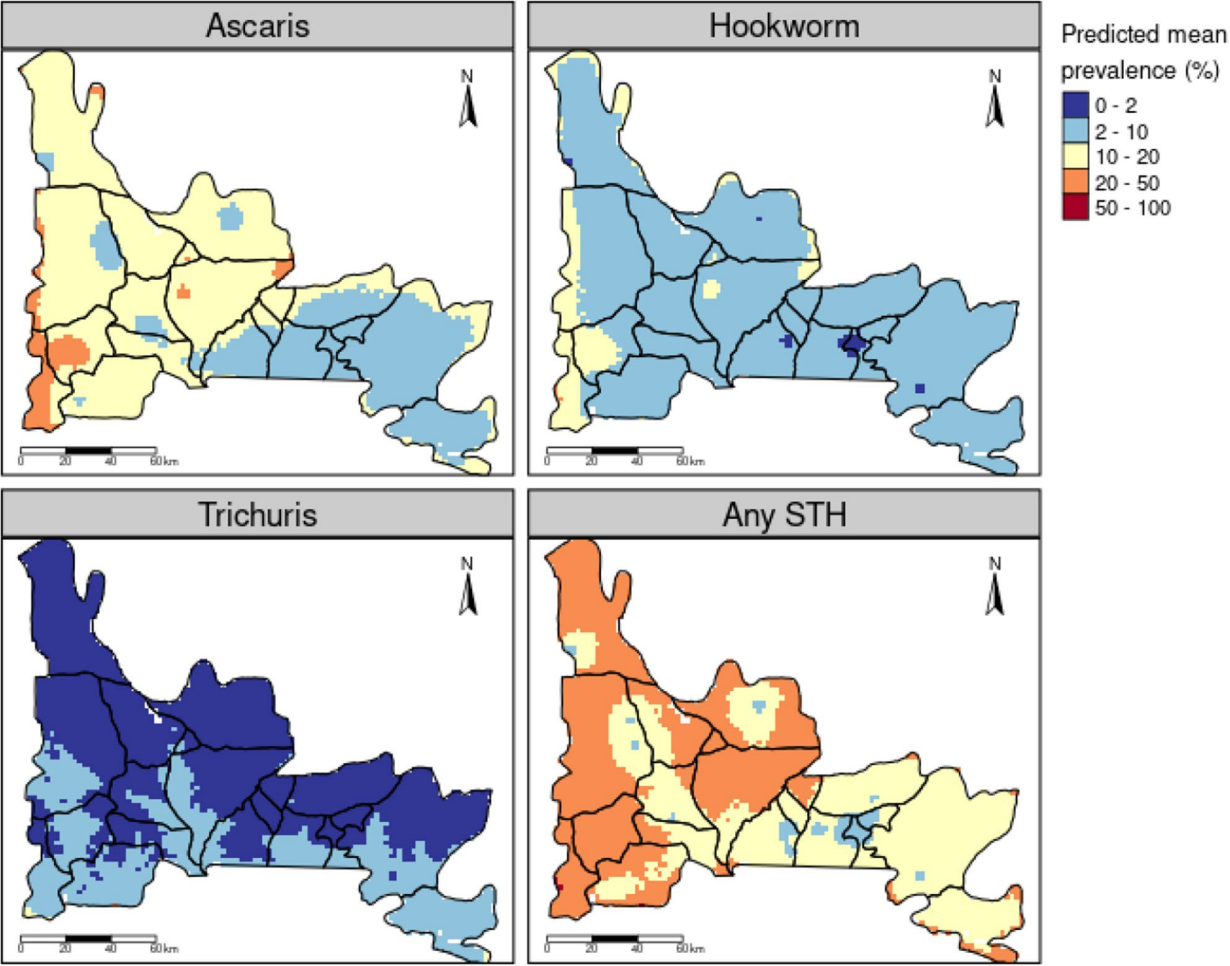
Map showing the predicted risk of soil transmitted helminth infections in Ogun State. This figure was created by the authors in R programming software (R version 4.1.2, Vienna, Austria). Available at https://www.R-project.org/.

Overall predicted mean prevalence of STH infections in Ogun State is 19.2% (95% CrI 1.82, 58.9) and ranges from 7.0 to 38% across the IUs. However, by species, predicted mean prevalence for *Ascaris lumbricoides* was 12.4% (95% CrI 0.98, 44.9), ranging from 5.0 to 23.8% across the IUs*.* For hookworms, the predicted mean prevalence was 6.2% (95% CrI 0.73, 22.5), and ranges between 2.0 and 14.5% across the IUs. The predicted mean prevalence of 1.9% (95% CrI 0.13, 8.84) was estimated for *Trichuris trichiura,* with a range between 0.1 and 5.7% across the IUs (Table 4). Figure 2 present the overall and species-specific predictive risk maps of soil-transmitted helminth infections. Predictive risk map for overall STH infection shows a high prevalence (> 20%) for LGAs in the central and western part of the state. Pockets of very high prevalence (> 40%) were also predicted for the LGAs around the boundary regions in the south-western part of the state. However, predicted prevalence were predominantly between 12 and 15% in the eastern part of the state. For *Ascaris lumbricoides*, pockets of high prevalence (> 20%) was predicted in the central and western part of Ogun State, with hotspots in the LGAs located close the border regions in the south-western part of the country. Moderate to high prevalence (5–20%) were also predicted in these regions. However, low predicted prevalence (5–10%) were observed in the eastern part of the country, with a sparse predicted prevalence within 10–12% around the border regions (Fig. 2). For hookworms, the predicted risk map shows that most regions have prevalence below 20%, except some pocket areas in Ipokia LGA, around the boundary lines. Predicted prevalence were predominantly between 5 and 10% in the central and western part of the state. However, predicted prevalence were lower (2–5%) in the eastern regions (Fig. 2). For *Trichuris trichiura* infections, most of the LGAs in the northern part of the state had predicted prevalence value between 0 and 1%. Similarly, in the southern region, predicted prevalence were predominantly between 5 and 10% (Fig. 2). The uncertainty of these estimates is presented in the standard error maps which can be found in Supplementary Fig. S1.

**Table 4:** Soil-transmitted helminth species-specific model-based predicted prevalence across the 20 LGAs in Ogun State, Nigeria.

LGA	*Ascaris lumbricoides*		Hookworms		*Trichuris trichiura*		Any STH	
	Mean prevalence (%)	95% CrI	Mean prevalence (%)	95% CrI	Mean prevalence (%)	95% CrI	Mean prevalence (%)	95% CrI
Abeokuta South	18.5	2.5, 54.2	9.6	2.2, 25.7	1.6	0.2, 5.5	27.5	4.8, 67.8
Abeokuta North	15.1	1.3, 51.6	7.7	0.9, 26.7	1.1	0.1, 4.5	22.4	2.3, 65.9
Ado-Odo Ota	12.4	0.7, 49.6	6.2	0.5, 26	3.1	0.3, 14.5	20.3	1.5, 67.4
Ewekoro	15.3	1.2, 52.5	8.1	0.7, 30.3	1.4	0.1, 6.5	23.1	2, 67.3
Ifo	17.5	1.7, 56.3	9.3	1.2, 31	2.3	0.2, 9.5	26.8	3.1, 72.5
Ijebu East	11.3	0.9, 41.7	5.3	0.6, 18.6	1.8	0.1, 7.4	17.5	1.6, 55.4
Ijebu Ode	11.2	0.6, 47.1	5.4	0.5, 23.4	3.1	0.1, 16.3	18.7	1.2, 65
Ijebu North	6.7	0.5, 26.9	2.7	0.4, 8.5	0.7	0.1, 2.4	9.8	1, 34.6
Ijebu North-East	8.7	0.4, 37.8	3.8	0.3, 16	2	0.1, 8.8	13.9	0.9, 51.8
Ikenne	8.5	0.6, 34.5	3.7	0.5, 13.7	0.8	0.1, 3.4	12.6	1.1, 44.7
Imeko-Afon	5	0.3, 22.2	2	0.2, 7.2	1	0.1, 4.3	7.7	0.5, 30
Ipokia	10.5	0.9, 38.2	4.7	0.8, 14.7	0.9	0.1, 3.3	15.4	1.7, 48.8
Obafemi-owode	14.6	1, 53	7.7	0.6, 30.7	0.1	0, 0.7	21.1	1.6, 66.4
Odeda	23.8	2.1, 71.2	14.5	1.5, 51	5.1	0.3, 25	38	3.9, 88.1
Odogbolu	14.9	1.3, 51.3	7.5	1, 25.6	2.5	0.2, 10.8	23.2	2.4, 67.7
Ogun waterside	15	1.1, 53.7	7.8	0.7, 30	0.4	0, 1.9	21.9	1.8, 67.3
Remo North	6.3	0.4, 27.6	2.5	0.3, 9.5	2	0.1, 8.6	10.5	0.8, 39.3
Shagamu	9.2	0.4, 43.3	4.4	0.3, 21.7	5.7	0.3, 28.8	18.1	1, 65.3
Yewa North	14.2	1.2, 49.7	7	1, 23.6	0.7	0, 3.3	20.8	2.2, 62.6
Yewa South	8.5	0.5, 36.5	3.8	0.4, 15.8	2.4	0.1, 11.3	14	1, 50.5
	12.36	0.98, 44.9	6.16	0.73, 22.5	1.94	0.13, 8.8	19.2	1.82, 58.9

### Estimates of number of people infected and annual drug requirements

Table 5 shows the number of infected individuals, estimated rounds of MDA and drug requirements for combined treatment of entire population and school-aged population across the IUs in the State. About 55% (11/20) of the IUs in the State requires biannual rounds of MDA, while 35% (7/20) and 10% (2/20) requires annual and biennial MDA rounds respectively. For the entire population, comprising of preschool-aged children, school-aged children and adults, a total of 1,099,461 persons are estimated to be infected, requiring 7,866,374 drugs. By IUs, about 10 LGAs has over 50,000 infected persons each, requiring more than 300,000 albendazole or mebendazole tablets. Ado Odo-Ota LGA has the highest number of infected population (168,591 persons) and requires 1,204,423 albendazole or mebendazole tablets. The least number of infected population (9103 persons) were estimated for Ijebu North-east, requiring 64,181 tablets.

**Table 5:** Estimates of number of people infected and annual drug requirements in Ogun State, Nigeria.

LGAs	Total population*			School-aged population	
	Rounds of MDA	Number infected (95% CrI)	Drug required (95% CrI)	Number infected (95% CrI)	Drug required (95% CrI)
Abeokuta South	Biannual	96,555 (16,734, 238,100)	702,821 (175,706, 1,054,233)	32,531 (5639, 80,218)	236,796 (59,200, 355,195)
Abeokuta North	Biannual	78,023 (8019, 228,140)	626,482 (119,234, 1,027,760)	26,304 (2703, 76,918)	211,211 (40,178, 346,516)
AdoOdo/Ota	Biannual	168,591 (13,254, 559,035)	1,204,423 (57,743, 2,446,759)	56,556 (4437, 187,567)	404,123 (19,332, 821,452)
Ewekoro	Biannual	16,261 (1541, 51,124)	112,703 (7651, 251,037)	5295 (502, 16,645)	36,703 (2500, 81,721)
Ifo	Biannual	144,387 (9163, 503,643)	962,756 (56,218, 2,259,466)	46,987 (2986, 163,857)	313,367 (18,346, 735,361)
Ijebu North-East	Annual	9103 (952, 31,813)	64,181 (0, 181,466)	3118 (326, 10,895)	21,980 (0, 62,147)
Ijebu East	Annual	23,919 (1575, 88,930)	170,024 (2125, 433,783)	8142 (534, 30,285)	57,833 (719, 147,608)
Ijebu North	Biennial	53,164 (4684, 188,646)	404,098 (463, 955,878)	18,392 (1620, 65,264)	139,790 (160, 330,666)
Ijebu Ode	Annual	17,256 (1108, 67,015)	129,554 (0, 399,428)	5735 (368, 22,273)	43,047 (0, 132,749)
Ikenne	Annual	25,255 (3005, 79,001)	158,432 (22,498, 401,004)	9383 (1117, 29,343)	58,859 (8399, 148,883)
Imeko-Afon	Biennial	25,379 (1872, 79,777)	205,578 (5469, 349,568)	9543 (704, 29,998)	77,292 (2059, 131,463)
Ipokia	Annual	79,081 (8309, 184,435)	438,345 (86,859, 630,572)	28,374 (2983, 66,162)	157,248 (31,170, 226,190)
Obafemi-Owode	Biannual	73,179 (7628, 213,975)	539,667 (77,337, 949,675)	24,032 (2505, 70,270)	177,229 (25,396, 311,887)
Odeda	Biannual	39,442 (3278, 121,247)	316,882 (32,924, 528,789)	13,508 (1120, 41,538)	108,502 (11,211, 181,054)
Odogbolu	Biannual	21,905 (1713, 82,002)	163,692 (2221, 455,374)	7888 (618, 29,504)	58,971 (811, 163,260)
Ogun Waterside	Biannual	19,173 (951, 69,839)	134,592 (2821, 303,131)	7182 (356, 26,161)	50,417 (1058, 113,550)
Remo-North	Biannual	17,235 (1839, 51,901)	146,251 (16,767, 248,217)	6242 (667, 18,797)	52,931 (6081, 89,937)
Shagamu	Annual	57,167 (4014, 206,168)	384,169 (5089, 1,044,285)	19,111 (1348, 68,879)	128,492 (1712, 349,374)
Yewa North	Biannual	60,549 (5210, 177,513)	458,509 (63,126, 762,141)	21,709 (1868, 63,641)	164,417 (22,648, 273,179)
Yewa South	Annual	73,838 (8555, 197,955)	543,215 (127,154, 815,251)	25,343 (2936, 67,936)	186,407 (43,634, 279,757)
Total		1,099,461(103,402, 3,420,260)	7,866,374 (861,403, 15,497,817)	375,374 (35,339, 1,166,152)	2,685,618 (294,614, 5,281,949)

*Total population comprises of preschool-aged children, school-aged children and adults; CrI : Credible interval.

However, for school-aged population, a total of 375,374 were infected, requiring 2,685,618 drugs for preventive chemotherapy. Ten LGAs in the central and western part of the state had over 10,000 infected school-aged children, and requires over 100,000 albendazole or mebendazole tablets each, for mass administration campaigns. The highest number of infected school-aged children (56,556) were estimated for Ado-Odo Ota LGA, requiring a total of 404,123 albendazole or mebendazole tablets. The least number of infected school-aged population (3118) were estimated for Ijebu North-east, requiring 21,980 tablets.

## Discussion

In this study, we utilized soil-transmitted helminth infection data from a state-wide cross-sectional survey to produce model-based estimates of infection risk, number of people infected, rounds of MDA and annual drug requirements for preventive chemotherapy. Empirical prevalence estimates for each of the three STH were below 20%, with *Ascaris lumbricoides* been the most predominant species (13.6%), followed by hookworm (4.6%) and *Trichuris trichiura* (1.7%)^19^. However, based on our model predictions, prevalence ranged from 5.0 to 23.8% for *Ascaris lumbricoides*, from 2.0 to 14.5% for hookworms, and from 0.1 to 5.7% for *Trichuris trichiura* across the IUs. However, location-specific predictions shows that overall STH and *Ascaris* infections were as high as 53% and 34% respectively, and greatest around the border of Republic of Benin in the west. Also, heavy risk approaching thresholds level necessitating preventive chemotherapy were observed in the central and western region. The risk of hookworms, also exhibit a similar pattern, however the predicted prevalence was further reduced below PC threshold levels. Majority of the LGAs were at very low risk of *Trichuris* infection. The spatial patterns observed in this study is in-line with the findings of^24^ for the three STH species, except for *Ascaris lumbricoides* where additional risk was reported in the eastern part of the state. This observation might be explained by the differences in composition of the study population (total population versus SAC only) and sampling point (communities versus school) in this present study^19,24^.

Furthermore, our results indicate the influence of some environmental covariates on transmission of soil transmitted helminth infections. For example, soil pH was negatively associated with *Ascaris*, suggesting that as pH of the soil increases, the survivability of *Ascaris* egg reduces. The effect of elevated pH on inactivation of Ascaris eggs have been previously reported^40^. Similarly, soil moisture was negatively associated with the risk of hookworm infection. This finding corroborates the observation of^41^. Temperature and moisture are determining factors in the development of helminth eggs^42^, with rainfall playing a major role in the restoration of the latter^43^. However, there are presumptions that heavy rainfalls might wash out soil transmitted helminth eggs from the soil^23,42,44^. This might explain the negative relationship between soil moisture and hookworm infections. Also, elevation was negatively associated with Trichuris. This supports already established evidence that the risk of *Trichuris trichiura* is rare or absent as altitude increases^45,46^. Our findings are also in-line with previous reports from Bolivia^47^ and Nigeria^24^.

STH infections thrives in areas lacking sanitation, potable water source, personal and domestic hygiene^4,7,9,19^, hence we expected infections to be associated with socio-economic predictors such as access to improved sanitation and drinking water facilities. However, none of the socioeconomic variables were picked during the geostatistical variable selection process. This finding is similar with those reported in Cambodia^22^, India^48^, Ethiopia^49^ and Australia^50^. Reasons for the no-effect association may not be limited to; (1) probable loss of variability as a result of household data aggregation for community analysis^22^, (2) insufficient coverage of water and sanitation resource facilities^48^, (3) Lack of standard and better water, sanitation and hygiene assessment tool leading to information bias^49^ and latrine efficiency in containing excreta^49^.

Prior efforts to model the treatment needs and number of SAC infected in Ogun State, were based on national survey data collected across 555 locations in Nigeria, with less than 20 location-specific data in Ogun State^24^. Indeed, these data may not reflect the actual situation of infected SAC and drug requirements for PC in the state^24^. Our study therefore presents, a robust estimate for the state, using more recent survey data collected across 1027 locations in the State. Based on our estimate, about 55% (11/20) of the IUs in the State requires biannual rounds of MDA, while 35% (7/20) and 10% (2/20) requires annual and biennial MDA rounds respectively. We therefore estimated a total of 1.1 million infected persons (comprising pre-school aged children, school-aged children and adults) and a total of 7.8 million albendazole or mebendazole tablets in Ogun State. More specifically, we estimate that 375,374 SAC were infected and a total of 2.7 million albendazole or mebendazole tablets will be required for PC. These estimates are twice as high as the number of tablets reported in^24^ for the state.

This study has shown the predicted prevalence using a robust geostatistical approach, and as well the spatial pattern of disease spread. The empirical and predicted prevalence for *Ascaris* infections were above 20%, hence necessitating annual PC in most regions. However, there were significant reduction in the prevalence and spread of Hookworm and *Trichuris* infections. This observation reflects the yields of investment made by the WHO, donor agencies, and various governmental and non-governmental health development agencies supporting PC in the country.

Our predictive maps and estimated drug requirements are therefore important in planning, targeting and delivery of prioritized interventions. The maps can also be utilized for designing more robust spatial surveys to meet more specialized needs including evaluation of STH control programs or long-term surveillance. Furthermore, we believe our estimations on the number of pre-school aged children and adults infected are useful, in the phase of expanding PC to adult population, to sustain accrued gains in morbidity control and interruption of transmission^51^.

## Conclusion

The work presented here contributes to the existing body of knowledge on model-based estimates of the geographical distribution of soil-transmitted helminth infection risk at more finite scale (i.e., scale smaller than the implementation units) in Ogun State in Nigeria. We used data generated across a community based cross-sectional study focusing on all sub-sets of a population (pre-school aged children, school-aged children and adults) to; (1) predict disease distributions, (2) identify associated environmental and socioeconomic risk factors, (3) estimate number of persons infected, and (4) estimate annual drug requirements. Our prediction maps provide useful information for identifying priority areas where interventions targeting soil transmitted helminthiasis are most urgently required. In addition, our estimations of drug needs are useful in the process of resource acquisition, planning and delivery of interventions.

## Supplementary Information

Supplementary Figures.

## Data Availability

The datasets for environmental and socio-economic variables are publicly available in the remote sensing data repositories cited within the text. The primary STH infection datasets analyzed for this study have also been previously published and are available at https://doi.org/10.1371/journal.pone.0233423.s001.

## Abbreviations

PC: Preventive chemotherapy
STH: Soil transmitted helminths
WHO: World Health Organization
SAC: School-aged children
GIS: Geographic information system
RS: Remote sensing
IU: Implementation units
LGAs: Local government areas
SAF: Sodium acetate acetic acid formaldehyde
NDVI: Normalized difference vegetation index
EVI: Enhanced vegetation index
LSTD: Land surface temperature for day
LSTN: Land surface temperature for night
MDA: Mass drug administration
NLE: Night light emission
MODIS/TERRA: Moderate resolution imaging spectroradiometer
C3S: Corpernicus climate change service
WORLDPOP: World population database
LBD: Local Burden of Disease Project
AIC: Akaike information criterion
GMRF: Gaussian Markov random field
INLA: Integrated nested Laplace approximation
SPDE: Stochastic partial differential equation
CI: Confidence interval
OR: Odd ratio
BCI: Bayesian credible interval
