# Supplementary material for: Estimating the population at risk with soil transmitted helminthiasis and annual drug requirements for preventive chemotherapy in Ogun State, Nigeria
Source: Sci Rep. 2022 Feb 7;12:2027. doi: 10.1038/s41598-022-06012-1 (PMC8821603; doi:10.1038/s41598-022-06012-1)
Supplement: Supplementary file 1 — Supplementary Figures. [file 41598_2022_6012_MOESM1_ESM.docx]

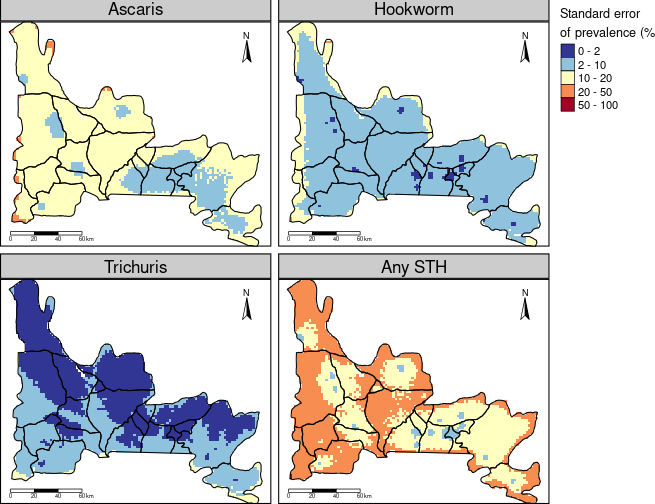
**Supplementary Figure S1: Map showing the standard error of the predicted risk of soil transmitted helminth infections in Ogun State. This figure was created by the authors in R programming software (R version 4.1.2, Vienna, Austria). Available at https://www.R-project.org/.**
